# Generation and utilization of polyfunctional anti-tumor CD4+ T cells

**DOI:** 10.1186/2051-1426-3-S2-P63

**Published:** 2015-11-04

**Authors:** Gang Zhou, Zhi-Chun Ding

**Affiliations:** 1 Georgia Regents University, Augusta, GA, USA

## Background

There is accumulating evidence that polyfunctional T cells, effector T cells capable of simultaneously producing multiple pro-inflammatory cytokines, are more efficacious in controlling infection and cancer. However, how polyfunctional CD4+ effector cells are induced is not mechanistically understood.

## Results

In this study we established that IL7 can promote the acquisition of polyfunctionality in naïve CD4+ T cells upon antigenic stimulation *in vitro*. In particular, IL7-conditioned polyfunctional CD4+ T cells can concomitantly express IFN-γ, TNF-α, IL-2 and granzyme B, with a separate IL4-producing population. We demonstrated that IL7 signaling resulted in increased histone acetylation in the promoters of effector molecules including IFN-γ, TNF-Αalpha, IL-2 and granzyme B, but not in Foxp3 and PD1, suggesting a selective enhancement in chromatin accessibility.

Mechanistically, STAT5 is required for IL7-driven polyfunctionality as expression of constitutive active STAT5 mutant in CD4+ T cells conferred polyfunctionality to CD4+ T cells even in the absence of IL7, whereas expression of dominant negative STAT5 mutant abolished IL7-driven polyfunctionality. Surprisingly, fully armed polyfunctional CD4+ T cells did not exhibit potent anti-tumor effect when adoptively transferred into mice with established B cell lymphoma, suggesting the dominance of immune suppression in the tumor microenvironment. Durable curative anti-tumor effect can be achieved by providing TriVax, a vaccine consisting of peptide, poly-IC adjuvant and OX40 antibody, following polyfunctional CD4+ T cell transfer.

## Conclusions

Our results provide novel insights into the generation of polyfunctional CD4+ effector cells and their potential usage in cancer immunotherapy.

